# Quantifying the treatment efficacy of reverse transcriptase inhibitors: new analyses of clinical data based on within-host modeling

**DOI:** 10.1186/1471-2458-9-S1-S11

**Published:** 2009-11-18

**Authors:** Romulus Breban, Sonia Napravnik, James Kahn, Sally Blower

**Affiliations:** 1 Semel Institute for Neuroscience and Human Behavior, David Geffen School of Medicine, University of California, Los Angeles, CA 90024, USA; 2 Unité d'Épidémiologie des Maladies Émergentes, Institut Pasteur, Paris, France; 3 School of Medicine, University of North Carolina, Chapel Hill, NC 27599, USA; 4 Positive Health Program, San Francisco General Hospital and the University of California, San Francisco, CA 94143, USA; 5 UCLA AIDS Institute, David Geffen School of Medicine, University of California, Los Angeles, CA 90024, USA

## Abstract

**Background:**

Current measures of the clinical efficacy of antiretroviral therapy (ART) in the treatment of HIV include the change in HIV RNA in the plasma and the gain in CD4 cells.

**Methods:**

We propose new measures for evaluating the efficacy of treatment that is based upon combinations of non-nucleoside and nucleoside reverse transcriptase inhibitors. Our efficacy measures are: the ***CD4 gain per virion eliminated***, the ***potential of CD4 count restoration and the viral reproduction number (R_0_)***. These efficacy measures are based upon a theoretical understanding of the impact of treatment on both viral dynamics and the immune reconstitution. Patient data were obtained from longitudinal HIV clinical cohorts.

**Results:**

We found that the ***CD4 cell gain per virion eliminated ***ranged from 10^-2 ^to 600 CD4 cells/virion, the ***potential of CD4 count restoration ***ranged from 60 to 1520 CD4 cells/*μ*l, and the basic reproduction number was reduced from an average of 5.1 before therapy to an average of 1.2 after one year of therapy. There was substantial heterogeneity in these efficacy measures among patients with detectable viral replication. We found that many patients who achieved viral suppression did not have high CD4 cell recovery profiles. Our efficacy measures also enabled us to identify a subgroup of patients who were not virally suppressed but had the potential to reach a high CD4 count and/or achieve viral suppression if they had been switched to a more potent regimen.

**Conclusion:**

We show that our new efficacy measures are useful for analyzing the long-term treatment efficacy of combination reverse transcriptase inhibitors and argue that achieving a low ***R_0 _***does not imply achieving viral suppression.

## Introduction

With currently available combination antiretroviral therapy (ART), the majority of patients achieve viral suppression within 24 weeks of initiation [1]. We hypothesize that further characterization of ART outcomes could differentiate among the vast majority of patients who achieve viral suppression but do not reach the immunologic reconstitution that matches their reduction in viral replication. Such characterizations may help further refine the guidelines for monitoring ART response.

Within-host HIV modeling has been a cornerstone for understanding HIV dynamics. Within this modeling paradigm, every patient is described by a set of fixed immune and viral parameters. The dynamics of HIV infection take place on two different timescales: fast viral and CD4 cell population dynamics that change on the timescale of months, and slower dynamics on the timescale of years that describe the decay of the patient's immune system. For the past decade, a vast amount of modeling work has been dedicated to understanding the interaction between the human immune system and HIV. Studies have been devoted to fitting models to within-host data and building models to provide both quantitative and qualitative answers. The principles of the within-host HIV fast dynamics are now relatively well-understood [2-6]. Further developments have focused on incorporating other elements of interaction between HIV and the immune system, such as cytotoxic T lymphocytes [4,7-9] and latently infected T cells [10-13].

Much effort has also been devoted to modeling the impact of treatment on the within-host HIV infection [2-4,14-26]. Major topics have been optimizing treatment for viral load reduction and CD4 increase [18-20], HIV drug resistance [15,16,24-26], adherence to therapy [15,16,20], structured treatment interruptions [21-23] and others. However, clinical applications of the understanding of fast dynamics have been limited because the necessary analyses, based upon these models, require detailed data that are difficult to obtain in large amounts from clinical trials or routine clinical care.

Here, we show how a mathematical model can be used to characterize a patient's response to a common ART regimen, the combination of nucleoside plus non-nucleoside reverse transcriptase inhibitors (NRTI/NNRTI). We use our model and novel data analysis techniques to analyze data from large longitudinal HIV clinical cohorts in order to characterize treatment efficacy. We quantify treatment efficacy by developing new surrogate markers for measuring ART outcomes. Specifically, we quantify the pace of immune destruction and the impact of therapy on the viral reproduction number. We discuss the implications of our analyses for clinical decision making.

## Materials and methods

### Patients and sampling

We analyzed data from a random group of 83 ART naïve patients receiving initial treatment with a NNRTI/NRTI regimen. Each patient had viral load and CD4 counts measured both before treatment and after approximately one year of treatment. Data were collected through the San Francisco General Hospital AIDS Program Database that was contained in the Healthcare Electronic Record Organizer (HERO) and from the UNC CFAR HIV Clinical Cohort Study. We defined the threshold of viral suppression to be 400 HIV RNA copies/ml.

### Mathematical methods

We consider a simple mathematical model that characterizes the fast viral dynamics of HIV infection

where *X *denotes the number of uninfected CD4 cells, *Y *denotes the number of infected CD4 cells and *V *denotes the infectious viral load. The parameters are as follows: *β *is the infectiousness of the HIV virus, 1/*δ *is the average lifetime of uninfected CD4 cells, 1/*α *is the average lifetime of infected CD4 cells, and *σ *is the ratio between viral load and the infected CD4 cells that we assumed to be constant. Our assumption is justified by the fact that the average lifetime of the virus in the blood is significantly shorter than either 1/*α *or 1/*δ *[3]. Thus, model (1) represents a good description of the within-host dynamics at timescales larger than the average lifetime of the virus, but smaller than the timescale of the destruction of the immune system. All the parameters in the model [equation (1)] given for a certain patient are expected to change only slowly with time, due to the slow destruction of the patient's immune system on the timescale of 10-15 years. We considered the model (1) for the interval of time of one to two years, so that the model's assumptions would hold. Such models of HIV dynamics have been previously used by Bonhoeffer and others [6,27]. The model is reduced to two dimensions when the third equation is used to replace the free-virus variable in the ordinary differential equations (ODEs) and express them only in terms of infected and uninfected CD4 cell populations. This approach has helped understand the foundations of HIV within-host dynamics [6,27]. However, the ODEs expressed in the CD4 population variables are difficult to interface with patient-specific clinical data that typically provide only the total CD4 count (*C *= *X*+*Y*) and the viral load (*V*) of the patient.

Here, we have thus adopted a different approach to a two-dimensional reduction of the model: we applied a change of variables in model (1) and obtained

The system described by model (2) has two time independent states (i.e., equilibria). One of them is the disease-free state (*C*_*h *_= *π*/*δ*, *V*_*h *_= 0) and the other is the viral set-point

**Figure 1:**
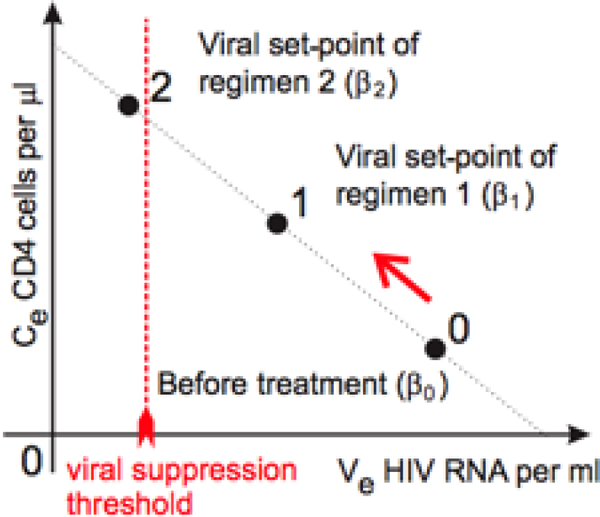
**Schematics of the theoretical linear relation between the endemic equilibrium (i.e., viral set-point) values of the CD4 count and the viral load of patients on NNRTI/NRTI regimens**.

Viral load is reduced in the presence of ART and a new viral set-point is reached. This effect is modeled by changing the model's parameters. Changing *β *mimics the effect of NNRTI/NRTI treatments [6,14], whilst changing *σ *mimics the effect of PI treatments. Thus, NNRTI/NRTI regimen types are modeled by changing only the parameter *β*, whilst NNRTI/NRTI and PI regimens necessitate changing both *β *and *σ*. In the case of NNRTI/NRTI therapy, there exists a linear relation between the total CD4 cell count, *C*_*e*_, and the viral load, *V*_*e*_, at the viral set-point during treatment; see Figure 1. We obtained this relation as follows. Using equation (3), we solved the *V*_*e *_formula for *α*/*β*, substituted the result in the *C*_*e *_formula and obtained

Equation (4) is independent of *β*, and describes the viral set-points that are reached during various NNRTI/NRTI regimens corresponding to various values of *β*.

Since equation (4) is linear, knowledge of the endemic equilibrium (i.e., the viral set-point) of a patient before treatment and during a particular NNRTI/NRTI treatment is enough to predict the patient's response to *all *NNRTI/NRTI treatments. Our model assumes that drug-naïve patients do not develop drug resistance before reaching their viral set-points during therapy. This assumption is reasonable since most patients reach their new viral set-point within one year of therapy [28]. Figure 2 of [28] shows clinical trial data on how the CD4 count varies with time under various NNRTI/NRTI regimens. The featured variation approaches a plateau after approximately one year of treatment. Analysis of the HIV RNA count is less reliable, as HIV patients become virally suppressed and their viral load is hard to measure accurately. It is important to note that our model can cope with certain patterns of slight non-adherence that do not accelerate the development of drug resistance [15,16]; non-adherence can be modeled by using an effective parameter *β *that is higher than the *β *value of the completely adherent patient.

HIV-infected drug-naïve patients have a high viral load and a low CD4 count at their viral set-point; see Figure 1. Due to treatment, their viral set-point shifts, and the patient reaches a new viral set-point with a lower viral load and a higher CD4 count. We used our model to analyze patient data from these two viral set-points: one pre-treatment viral set-point and one following a year of NNRTI/NRTI treatment. The slope of the CD4-viral load graph (i.e., a graph obtained from equation 4) gives a measure of how each patient's immune system could recover if the virus were eliminated. Mathematically, the slope, s, is specified by

and its magnitude, |*s*|, represents the CD4 gain per virion eliminated.

**Figure 2:**
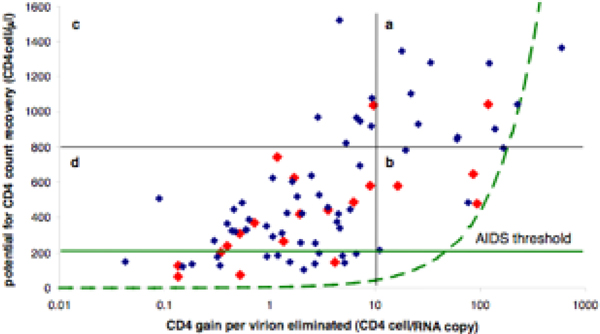
**Graph of the potential for CD4 count recovery (i.e., intercept) versus the CD4 gain per virion eliminated (i.e., the magnitude of the slope)**. Notice that the patients split into four categories: (a) patients with a high slope magnitude and high intercept, (b) patients with a high slope magnitude and low intercept, (c) patients with a low slope magnitude and high intercept, and (d) patients with a low slope magnitude and low intercept. The red dots represent patients that did not reach viral suppression after one year of therapy. The continuous green line marks the AIDS threshold. The dotted green curve marks the set of parameters for which individuals reach viral suppression (i.e., *V*_*e *_= 400 HIV RNA copies/ml) and low *R*_0 _(i.e., *R*_0 _= 1.1) simultaneously. Patients with parameters in the region to the right of the dotted curve would first reach a low *R*_0 _and then viral suppression while the converse holds to the left of the dotted curve. For very potent regimens, both viral suppression and low *R*_0 _are achieved by patients with parameters throughout this space.

The intercept, *i*, is specified by

Note that the intercept satisfies *i *= *C*_*h*_, which is the CD4 cell count of the disease-free state. Therefore, the intercept identifies the maximum CD4 count that a patient could attain if the virus were eliminated (i.e., the potential for CD4 count reconstitution). We quantified the patient responses to NNRTI/NRTI therapy by calculating the slope *s *and the intercept *i *of the CD4/viral-load linear relation for each patient. We also assessed a third efficacy measure, the basic reproduction number *R*_0 _[29]. For our model, *R*_0 _is given by

This formula was obtained through stability analysis of the uninfected state and provides a threshold parameter for signaling the spread of HIV infection in patients. We can justify the biological meaning of this *R*_0 _formula by constructing a Crump-Mode-Jagers branching process as an individual level model which yields the same predictions as our model given by equation (1). For such constructs, see [30] and [31]. *R*_0 _represents the average number of HIV virions produced by an infected CD4 cell that succeed in infecting other CD4 cells in the case when all CD4 cells were uninfected. Thus, *R*_0 _can be used to assess the severity of the infection. For a given patient, if *R*_0 _< 1, the infection will die out, but if *R*_0 _> 1 the infection will increase to a viral set-point. Since *R*_0 _depends on *β*, we can calculate two different values for *R*_0 _(i.e., one for the viral set-point pre-therapy and one for the viral set-point after one year of therapy). Using equations (3) and (6), we rewrote the expression for *R*_0 _[equation (7)] in terms of the data which were available from the two viral set-points:

where *C*_*e *_is the CD4 count at the viral set-point, *i *is the intercept of the linear relation between the two viral set-points and *δ*/*α *is a dimensionless parameter that represents the ratio of the lifetime of an infected CD4 cell to the lifetime of an uninfected CD4 cell. This dimensionless parameter is not available from the viral set-point data. However, literature estimates provide 1/*δ *> 50 days [17] and 1/*α *H 2.2 days [3], thus leading to *δ*/*α *< 0.045. Since *δ*/*α *is small, we expanded the *R*_0 _formula with respect to this parameter:

The first term of this expression has been proposed as an *R*_0 _formula by Anderson and May (see [29], equation (2.1), pages 17-19) in an elementary host-parasite model:

Here, we accounted for the fact that infected and uninfected CD4 cells have different lifetimes and obtained a small correction to this formula. It may be argued that, in general, *R*_0_^*AM *^represents the bulk of the numerical estimate of *R*_0 _for many models where additional factors (e.g., different lifetimes for infected and uninfected CD4 cells, latently infected cell populations, preferential infection of activated and HIV-specific T cells [11-13,32-34]) bring relatively small corrections. Thus, equation (9) acquires a special status of generality and great practical importance. To estimate patient-specific *R*_0 _values, we used equation (9) where the second term in the expansion over *δ*/*α *represented an estimate of the modeling error.

It is important to note that becoming virally suppressed and achieving an *R*_0 _close to 1 are two independent conditions that do not imply each other. We now explain in detail why this is the case. First, let us consider a target *R*_0 _value that is close to one, *R*_0_*, which helps us formulate a condition for the patient being close to the elimination of the infection (i.e., condition of reduced viral dynamics)

Second, choosing a viral suppression threshold *V*_*s*_, the viral suppression condition is simply

However, writing *s *as

solving for *V*_*e*_, and using the fact that

equation (11) becomes

(Note that *s *is always negative.)

Analyzing equations (10) and (12), we obtain that, depending on his/her measures of NNRTI/NRTI treatment efficacy *i *and *s*, and the potency of the regimen, a patient may achieve both viral suppression and low *R*_0_, one or neither of these conditions. In particular, when

*R*_0_* < 1/[1+*V*_*s*_/(*i*/*s*)]: The patient may not have a low *R*_0_, yet achieve viral suppression for some NNRTI/NRTI treatment regimens.

1/[1+*V*_*s*_/(*i*/s)] <*R*_0_*: The patient may not be virally suppressed, yet have a low *R*_0 _for some NNRTI/NRTI treatment regimens.

*R*_0_* = 1/[1+*V*_*s*_/(*i*/*s*)]: In this case low *R*_0 _and viral suppression imply each other. However, these patients represent special cases and their values of NNRTI/NRTI treatment efficacy measures are related by the following linear relation

which divides the (*i*, |*s*|) space in the two regions specified above. (For *V*_*s *_= 400 HIV RNA copies/ml and *R*_0_* = 1.1, we obtain [*V*_*s*_/(1-1/*R*_0_*)] = 4400 HIV RNA copies/ml. Regions 1) and 2) are to the left and to the right of the dotted green curve in Figure 2, respectively.)

We emphasize that all patients are able to achieve both viral suppression and a low *R*_0_, provided that the NNRTI/NRTI treatment regimen is potent enough. The situations described above may occur for NNRTI/NRTI treatment regimens of intermediate strength.

## Results

**Figure 3:**
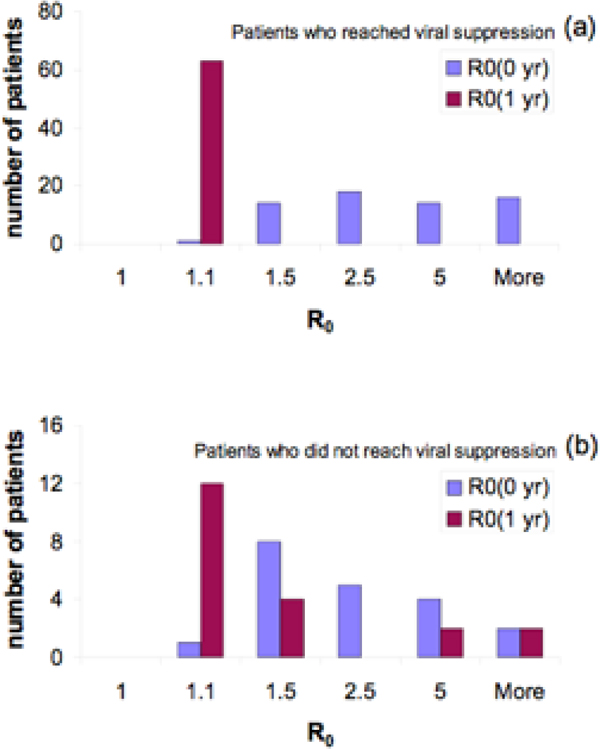
**(a) The histograms of the basic reproduction ratios, R_0_, before and after one year of treatment for patients who reached viral suppression**. (b) The histograms of the R_0 _values before and after one year of treatment for patients who did not reach viral suppression.

We analyzed data from a random sample of 83 ART-naïve patients from two HIV clinical cohorts. All patients initiated a NNRTI/NRTI regimen with measured plasma HIV RNA values and CD4 cell counts before treatment and after approximately one year of treatment. We used these data to calculate two distinct *R*_0 _values: one *R*_0 _value before treatment was initiated and one value after one year of treatment. Our calculated formula shows that *R*_0 _is a function of the potential for CD4 count reconstitution. We calculated that the *R*_0 _before treatment was initiated had an average value of 5.1 and, after one year of combination NNRTI/NRTI therapy, the average *R*_0 _had decreased to 1.2. In Figure 3 we show the frequency distributions of *R*_0 _before treatment was initiated (blue bars) and after one year of treatment (purple bars) for the patients who reached viral suppression (panel (a)) and for the patients who did not reach viral suppression (panel (b)). A comparison between panels (a) and (b) of Figure 3 reveals that all patients who attained viral suppression reached *R*_0 _< 1.1 after one year of treatment (Figure 3(a)). However, it can be seen that there are patients who do not achieve viral suppression, but, nevertheless, reached *R*_0 _< 1.1 after one year of therapy (Figure 3(a)). We estimated that the modeling errors in our calculated values of *R*_0 _are no larger than 1%. To our knowledge, this is the first time that the value of the basic reproduction number (*R*_0_) for HIV for an individual patient has been calculated from patient clinical care data.

**Figure 4:**
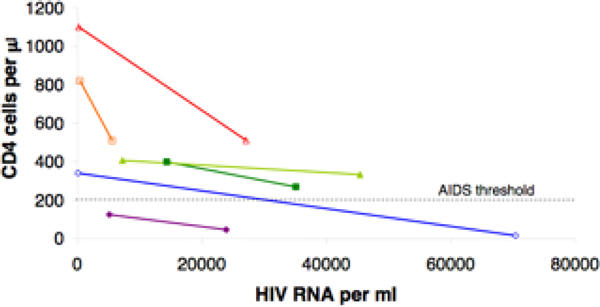
**Examples of patient response to NNRTI/NRTI treatment**. The right end of a line segment represents the viral set-point of the patient before treatment and the left end represents the viral set-point during treatment.

In order to calculate our two other efficacy measures, we calculated the slope and the intercept of the relation between CD4 count and viral load at two viral set-points for each patient; see Figure 4. We found that the *CD4 gain per virion eliminated *(i.e., the magnitude of the slope) ranged from 10^-2 ^to 600 CD4/virion and the *potential for CD4 count reconstitution *(i.e., the intercept) ranged from 60 to 1520 CD4/*μ*l. The average intercept (464 CD4 cell/*μ*l) was lower than the average CD4 count of a non-infected individual. Thus, our results show that, even if all replicating virus were eliminated, the CD4 cells of these patients would not be able to return to a normal level. Calculating the potential for CD4 count reconstitution, we are thus able to quantify the slow immune destruction which individuals undergo since HIV infection.

For a better illustration of the values that we found for our efficacy measures, we graphed the potential for CD4 count reconstitution versus the CD4 gain per virion eliminated; see Figure 2. A fair amount of correlation is observed between the potential for CD4 count reconstitution and the logarithm of the CD4 gain per virion eliminated (Pearson correlation coefficient ~0.675). Patients could be divided into four categories: (a) patients with a potential for both a high CD4 count reconstitution and a high CD4 gain per virion eliminated, (b) patients with a potential for only a low CD4 count reconstitution but a high CD4 gain per virion eliminated, (c) patients with a potential for a high CD4 count reconstitution but only a low CD4 gain per virion eliminated, and (d) patients with a potential for only a low CD4 count reconstitution and a low CD4 gain per virion eliminated. Surprisingly, many patients who attained viral suppression did not have high CD4 cell recovery profiles (the blue dots in the regions (b) and (d) of Figure 2). The other data (red dots) in Figure 2 also show substantial heterogeneity in our efficacy measures among the patients who were not virally suppressed. The red points are spread throughout the plane of potential of CD4 count reconstitution - CD4 gain per virion eliminated. This spread indicates that our new efficacy measures are not correlated with viral suppression. Most importantly, Figure 2 also shows that there was a subgroup of patients who were not virally suppressed but had the potential to reach a high CD4 count and/or achieve viral suppression if they had been switched to a more potent regimen (the red dots in region (a)).

Our results also imply that NNRTI/NRTI treatment regimens can have substantial impact on reducing viral dynamics (i.e., the *R*_0_) and that this effect can occur even in patients who do not seem to be responding well to treatment. This paradoxical situation may occur for individuals who have low CD4 gain per virion eliminated and high potential of CD4 count reconstitution. In particular, it is possible that, due to treatment, a patient does not reach viral suppression yet his/her CD4 count very much approaches his/her potential of CD4 count reconstitution, implying an *R*_0 _close to 1. Using our model, we predict that patients with NNRTI/NRTI treatment efficacy measures in the region which is to the left of the dotted green curve in Figure 2 may have an *R*_0 _close to 1 yet will nevertheless not reach viral suppression. The patients with treatment efficacy measures in the complementary region may achieve viral suppression yet maintain a high *R*_0 _which places them far from the elimination of the infection. In our case, all patients that become virally suppressed reach a low *R*_0 _(Figure 3(a), blue dots in Figure 2). However, we identify a group of 12 patients that do not become virally suppressed although they reach a low *R*_0 _and reduced viral dynamics (Figure 3(b)). All of them are placed to the left of the dotted curve in Figure 2.

## Discussion

We restricted our analyses to patients receiving the common ART combination of nucleoside plus non-nucleoside reverse transcriptase inhibitors. We concentrated on this treatment regimen for two major reasons. Firstly, NRTI plus NNRTI regimens are likely to remain popular as first-line therapy because of their demonstrated efficacy, simplicity of therapy, pill number and tolerability [1]. Secondly, NNRTI/NRTI therapy is likely to remain the dominant regimen for first-line therapy in resource-constrained countries. Among the 42 antiretroviral products whose price the Clinton foundation negotiated for resource-constrained countries only two contain PIs [35]. There are 71 countries (including the vast majority of sub-Saharan countries) that can now benefit from this negotiation, representing more than 92% of the people living with HIV globally [36,37]. It is important to note that, as a general trend, PIs are likely to remain prohibitively expensive for resource-constrained countries in the short term [38]. In fact, in their third annual report on the current rollout of treatment in Africa, PEPFAR cautions against poor management of first-line NNRTI/NRTI treatment regimens that would require an early introduction of more costly PI-based second-line regimens [39]. PEPFAR reports that only 10% of the treatment regimens that they currently sponsor in Africa are second line [39]. Therefore, it is expected that NNRTI/NRTI treatment regimens will remain the first and most important line of defense against the HIV pandemic in resource-constrained settings.

The need for developing new accurate and reliable surrogate markers for evaluating the clinical efficacy of antiretroviral agents has become a major focus of HIV clinical care. Surrogate markers have been used by regulatory agencies to approve new agents, by consensus panels to develop clinical guidelines and by investigators to determine clinical efficacy. In this paper, we have presented new methodologies for generating surrogate marker data in order to quantify new measures of treatment efficacy. Unlike previous efficacy measures, our efficacy measures are based upon a theoretical understanding of the impact of treatment on both viral dynamics and the immune response. We have shown that our methodology can be used to analyze data collected during routine clinical care. The advantage of our surrogate markers for measuring treatment efficacy is that they are patient-specific in contrast to the surrogate markers that have been developed previously from aggregate clinical trial data. Thus, we found that two of our surrogate markers have a moderate degree of correlation indicating that a low CD4 gain per virion eliminated may be associated with a low potential for CD4 count reconstitution. We have also developed for the first time a methodology for calculating patient-specific *R*_0 _estimates and used these values to quantify the efficacy of the NNRTI/NRTI treatment therapy. Thus, we showed that achieving a low *R*_0 _does not imply achieving viral suppression. Our new efficacy measures have also shown two important new results that have significant clinical implications. Our efficacy measures enabled us to identify a subgroup of patients who achieved viral suppression, but did not have a high likelihood of achieving a high CD4 cell count. Most importantly, our efficacy measures enabled us to identify a subgroup of patients who were not virally suppressed, but had the potential to reach a high CD4 count and/or achieve viral suppression if they had been switched to a more potent regimen.

## Conclusion

Based upon a theoretical understanding of the impact of HIV treatment on viral dynamics and immune reconstitution, we propose new measures for evaluating the efficacy of treatment with reverse transcriptase inhibitors. Our efficacy measures are: the CD4 gain per virion eliminated, the potential of CD4 count restoration and the viral reproduction number (R_0_). We show that our new efficacy measures are useful for analyzing the long-term treatment efficacy of combination reverse transcriptase inhibitors and argue that achieving a low R_0 _does not imply achieving viral suppression.

## Competing interests

The authors declare that they have no competing interests.

## Authors' contributions

RB, JK, SN and SB contributed to the design of the project, the interpretation of the results, and the writing of the manuscript. JK and SN contributed the necessary data for the project. RB performed the data analyses. All authors read and approved the final version of the manuscript.
